# Max Bergmann lecture Protein epitope mimetics in the age of structural vaccinology‡

**DOI:** 10.1002/psc.2482

**Published:** 2013-01-24

**Authors:** John A Robinson

**Affiliations:** Chemistry Department, University of ZurichWinterthurerstrasse 190, 8057, Zurich, Switzerland

**Keywords:** peptide, hairpin conformation, antibiotic, LptD, outer membrane, vaccine, HIV-1, virus-like particle

## Abstract

This review highlights the growing importance of protein epitope mimetics in the discovery of new biologically active molecules and their potential applications in drug and vaccine research. The focus is on folded *β*-hairpin mimetics, which are designed to mimic *β*-hairpin motifs in biologically important peptides and proteins. An ever-growing number of protein crystal structures reveal how *β*-hairpin motifs often play key roles in protein–protein and protein–nucleic acid interactions. This review illustrates how using protein structures as a starting point for small-molecule mimetic design can provide novel ligands as protein–protein interaction inhibitors, as protease inhibitors, and as ligands for chemokine receptors and folded RNA targets, as well as novel antibiotics to combat the growing health threat posed by the emergence of antibiotic-resistant bacteria. The *β*-hairpin antibiotics are shown to target a *β*-barrel outer membrane protein (LptD) in *Pseudomonas* sp., which is essential for the biogenesis of the outer cell membrane. Another exciting prospect is that protein epitope mimetics will be of increasing importance in synthetic vaccine design, in the emerging field of structural vaccinology. Crystal structures of protective antibodies bound to their pathogen-derived epitopes provide an ideal starting point for the design of synthetic epitope mimetics. The mimetics can be delivered to the immune system in a highly immunogenic format on the surface of synthetic virus-like particles. The scientific challenges in molecular design remain great, but the potential significance of success in this area is even greater. Copyright © 2013 European Peptide Society and John Wiley & Sons, Ltd.

## Introduction

Protein epitope mimetics (PEMs) are rapidly gaining prominence as a source of novel leads in drug and vaccine research. PEMs are designed to mimic the three-dimensional (3D) surface regions of peptides and proteins recognized by biological receptors. Considerable effort has focused already on the design of PEMs as potential inhibitors of protein–protein and protein–nucleic acid interactions [1]. However, exciting opportunities are now also arising for the use of PEMs in the structure-based design of synthetic vaccines, targeting a wide range of infectious diseases and chronic human health problems such as allergies, Alzheimer's, and cancer. Research on epitope mimetics has been driven forwards over the past decade by progress in high-throughput genomics and proteomics, as well as by the massive growth in the 3D structural database of biological macromolecules and the complexes they form. This is exemplified by the rapid recent growth in the number of crystal structures of antibody fragments derived from antibodies that protect against infection by an invading pathogen, bound to their pathogen-derived antigens (*vide infra*). Knowing at a structural level how antibodies recognize protective epitopes on pathogens heralds a new era of structural vaccinology, where this information can be exploited in rational structure-based approaches to vaccine design [2]. Nevertheless, it still remains a considerable scientific challenge to transform this 3D structural information into rationally designed molecules with the desired chemical and biological properties.

A crystal structure may reveal the surface shape and complementarity of protein–protein interfaces but not the energetic origins of binding affinity and specificity. Part of the problem lies in identifying the energetically important interactions that influence the stability of protein–protein and protein–ligand complexes. An important advance came with the identification in many protein–protein interfaces of a select group of ‘hot-spot’ residues that typically make a disproportionately large contribution to binding energy and can be easily identified by alanine-scanning mutagenesis [3]. The ‘hot’ residues often cluster on each surface at the center of interfaces, typically constitute less than half of the contact surface, and are often surrounded by other surface residues that appear to contribute relatively little in binding energy [4–6]. Not surprisingly, hot spots have become a major focus of interest in protein–protein interaction inhibitor design [7]. Double-mutant cycle analyses have revealed more recently that many protein–protein interfaces appear to be built in a modular fashion, with clusters of residues on each side involved in networks of strong intracluster interactions but with weak intercluster connections [5,8–10]. Within a small group of test protein complexes, the size and extent of cooperativity of interactions within a network cluster, rather than the surface area of the interface *per se*, seemed to correlate with higher binding affinity [10]. These and other studies have revealed that a higher network organization of interactions can occur at protein interfaces, as well as in protein–ligand and enzyme–inhibitor interactions, which may be important in accounting for ligand binding affinity and specificity [11,12]. Finally, the relationship between protein structure and the internal dynamics is also important for ligand binding. Although for most proteins, internal dynamics are only poorly or remain uncharacterized, new results suggest that both fast and slower internal dynamics can influence ligand binding and in ways that are difficult to predict from only the protein's ground state structure [13]. Collectively, these insights suggest limitations to the ‘knobs-into-holes’ approach, based upon maximizing surface complementarity, which is commonly taken in protein–ligand design studies.

**Biography**

**John Robinson** studied chemistry at the University College, London, where he was awarded the B.Sc. degree in 1974. He then completed a Ph.D. at Cambridge University in 1977. With a Royal Society postdoctoral fellowship, he subsequently carried out postdoctoral work in the Biochemistry Institute of the University of Karlsruhe, before joining the Chemistry Department of Southampton University in 1979 as a lecturer. He moved to Zurich as a full professor of organic chemistry in 1989.

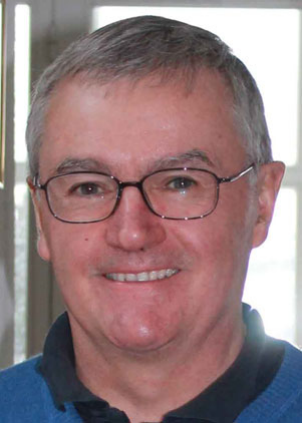

## *β*-Hairpin Epitope Mimetics

**Figure 1:**
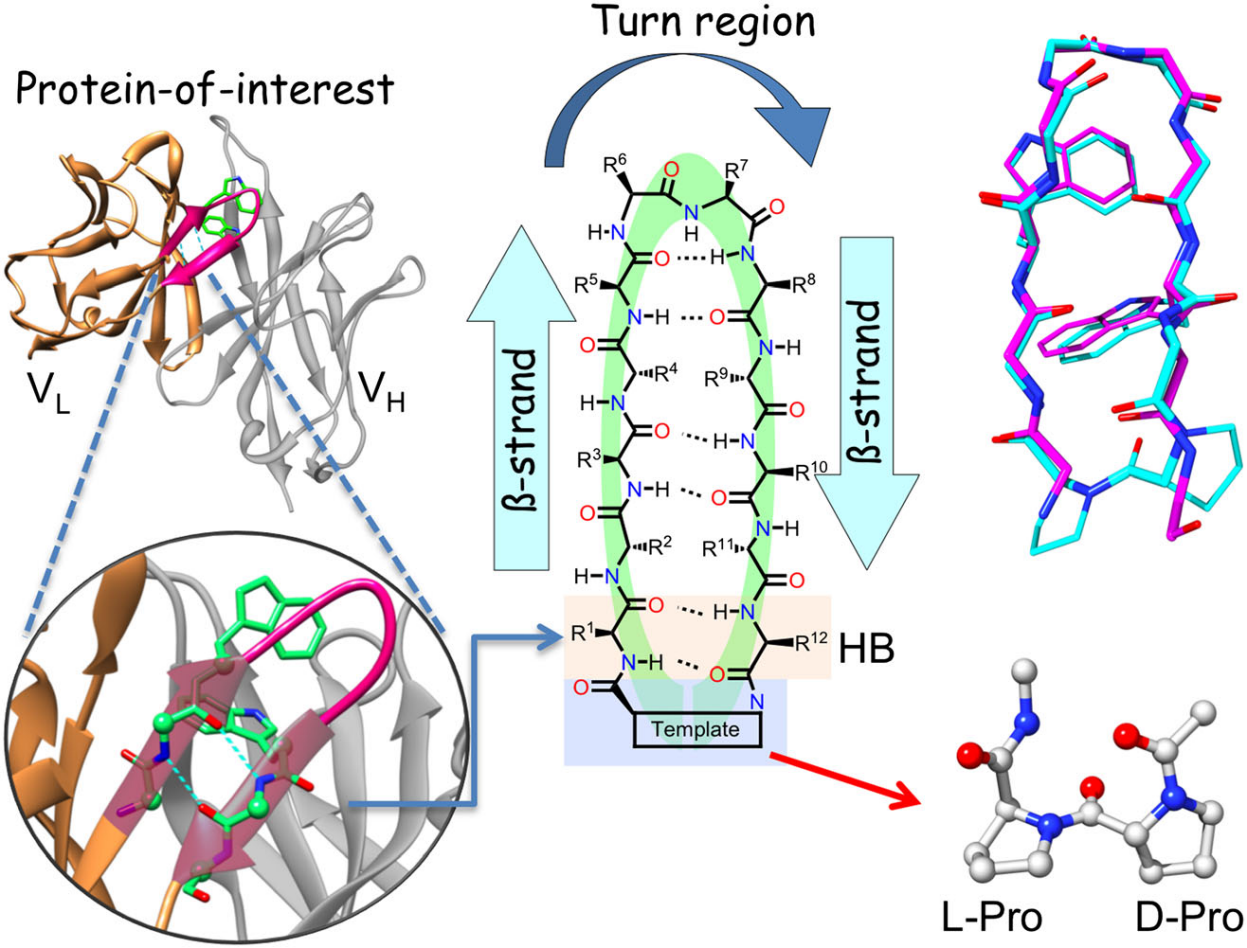
A *β*-hairpin loop identified in a protein crystal structure (*left*) can be transplanted onto a d-Pro- l-Pro template (*right*), resulting in cyclic *β*-hairpin mimetic (*center*). The template in the mimetic helps to stabilize folded *β*-hairpin conformations and fixes the hairpin register. A comparison of one NMR structure of a CDR loop mimetic (blue) with the same loop in the protein crystal structure is shown (*right*) [20].

Much of the work reviewed here has been focused upon the design of *β*-hairpin mimetics, based on motifs seen in protein crystal structures. *β*-Hairpin motifs are often found in proteins to mediate protein–protein and protein–nucleic acid interactions. A *β*-hairpin is comprised of two consecutive hydrogen-bonded antiparallel *β*-strands connected by a loop sequence. Many variations are observed in backbone conformation in *β*-hairpin loops in folded proteins, depending upon the hydrogen bonding (HB) pattern between the two antiparallel *β*-strands and the loop length (for reviews, see [14,15]). This structural diversity can be captured, at least to some extent, in *β*-hairpin mimetics designed by transplanting the hairpin loop from a protein of known structure onto a semirigid hairpin-stabilizing template, to afford a macrocyclic, conformationally constrained, template-bound *β*-hairpin mimetic [16]. The position of backbone cyclization, the conformational bias imposed by the constraining template, and the influence of the hairpin loop length and sequence can all influence the conformational stability of the folded *β*-hairpin structures. One very convenient template to use is the dipeptide d-Pro- l-Pro, which itself is known to adopt a stable type II′ *β*-turn [17–19]. When incorporated into cyclic peptide mimetics, this template nucleates *β*-hairpin conformations possessing the preferred right-handed twist typically observed between adjacent antiparallel *β*-strands in proteins. An early example of this approach was the design of hairpin mimetics based upon *β*-hairpin complementarity-determining region (CDR) loops in the antigen-binding site of antibodies (Figure 1). Excellent structural mimicry was observed between several cyclic hairpin mimetics and the corresponding CDR loops in antibody Fab fragments [20]. In the design of mimetics, it is important to recognize that paired cross-strand residues on opposite *β*-strands can exist at HB and non-HB (NHB) positions, and their side chains point to different sides of the hairpin [14,21]. When a *β*-hairpin loop is transplanted from a known protein structure onto d-Pro- l-Pro, the template must be inserted at an NHB position. The N-terminal and C-terminal loop residues will then be forced into an HB position, and the ensuing HB pattern should be maintained along the hairpin (Figure 1). Lengthening a loop, by inserting one residue at the C-terminus, causes all the residues in HB positions along this strand to move into NHB positions, and vice versa, thus altering the distribution of side chains on the two sides of the hairpin dramatically. This effect was observed in a series of *β*-hairpin mimetics based upon the V3 loop from the HIV-1 envelope glycoprotein gp120, as discussed in detail in the following [22]. The template, therefore, stabilizes the hairpin backbone conformation and fixes the hairpin register.

**Figure 2:**
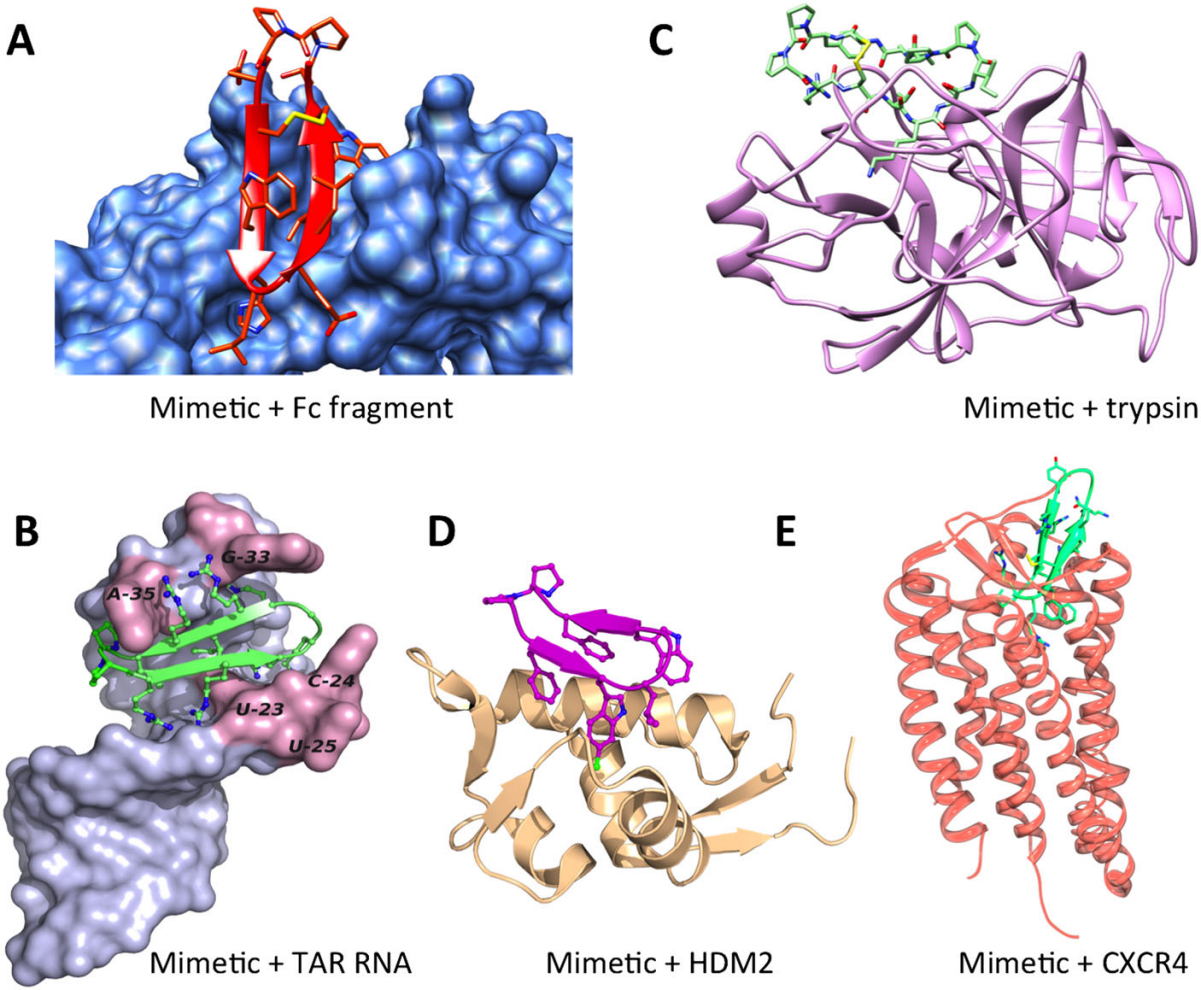
*β*-Hairpin mimetics have been discovered that bind with high affinity to the targets shown. The complexes with the Fc fragment (A) and trypsin (C) are computer models based upon crystal structures of target-bound phage (1DN2) or natural product (1SFI) leads. The complexes shown with TAR RNA (2KDQ) (B), HDM2 (2AXI) (D), and CXCR4 (3OE0) (E) are crystal or NMR structures, available in the Protein Data Bank database.

Another *β*-hairpin mimetic was based upon a disulfide-bonded phage display peptide that binds to the Fc region of an antibody. A crystal structure of the phage peptide bound to the Fc protein revealed a *β*-bulge in one of the *β*-strands [23]. A *β*-bulge occurs when two residues on one strand lie opposite a single residue on the other strand. *β*-Bulges affect not only the directionality of the backbone but also and more dramatically the orientation of side chains with respect to the *β*-hairpin plane. This feature was also observed in a *β*-hairpin mimetic derived from the phage display peptide (Figure 2A) [24]. The *β*-bulge places side chains of two consecutive residues (Val^10^–Trp^11^) onto the same side of the hairpin, where they can both interact with the surface of the target Fc protein.

Most *β*-hairpins in proteins of known 3D structure have loops of ≤5 residues [14]. In two residue hairpin loops, type I′ and II′ *β*-turns are strongly favored over type I and type II *β*-turns. A type I′ *β*-turn was observed at the tip of a 12-residue *β*-hairpin mimetic derived initially from the Tat protein, which binds to a nucleic acid target, the transactivation response element (TAR) RNA of HIV-1 [25,26]. The mimetic was found to adopt stable *β*-hairpin structures in free solution and when bound in the major groove of the TAR RNA hairpin (Figure 2B). In the RNA–peptidomimetic complex, side chains on both sides of the peptide hairpin are seen to make intimate contacts with the RNA, involving both hydrophobic and polar electrostatic interactions [26]. The tip of a *β*-hairpin may also contain larger loops [14]. A greater propensity for the occurrence of *cis*-peptide bonds is observed in four-residue and five-residue loops, involving Xaa-Pro peptide bonds in type VI *β*-turns. *cis*-Peptide bonds have been observed in a family of *β*-hairpin mimetics derived from a sunflower seed trypsin inhibitor (Figure 2C). For example, both 11-residue and 7-residue cyclic mimetics were shown to adopt a stable backbone hairpin fold with a five-residue loop and a stable *cis* Ile–Pro peptide bond at the tip of the hairpin. Both mimetics were also potent trypsin inhibitors [27].

An attractive feature of such *β*-hairpin mimetics is their ease of synthesis. Typically, a linear precursor can be assembled by solid-phase peptide chemistry and then cyclized in solution and deprotected. This assembly process is robust and amenable to parallel synthesis methods, allowing the production of small libraries of hairpin mimetics, for example, containing sequence variations [28]. Proteinogenic and nonproteinogenic amino acids, as well as an array of related building blocks, can be used for synthesis as a means to tailor and optimize the structure and biological properties of a mimetic. In this way, a family of *β*-hairpin peptides that can mimic an *α*-helical epitope in the p53 protein and bind with high affinity to its interaction partner, the HDM2 protein (Figure 2D) [29–31], was identified. In other examples, a family of *β*-hairpin mimetics that bind with high affinity and specificity to the chemokine receptor CXCR4 (Figure 2E) [32] was discovered, and yet another was found to bind and inhibit the bacterial *β*-barrel outer membrane (OM) protein LptD (discussed in detail later [33]). *β*-Hairpin mimetics, therefore, based upon folded *β*-hairpin motifs found in naturally occurring peptides and proteins, appear to represent an interesting source of novel ligands, with obvious potential for applications in drug and vaccine research [34,35].

## *β*-Hairpin Antibiotics Derived from Peptides of the Innate Immune System

Some very interesting naturally occurring *β*-hairpin-shaped peptides are found within the large family of cationic antimicrobial peptides (CAPs), which play important roles in innate immunity in many different organisms [36–40]. CAPs are produced in vertebrates (including humans), where they often provide a first line of defense against bacterial and viral infections. They typically display a fascinating and complex spectrum of biological activities. Many show broad-spectrum antimicrobial activity against Gram-positive and Gram-negative bacteria, as well as antiviral activity. In addition, many are known to exert complex immunomodulatory effects in animals, the mechanisms of which are still rather poorly characterized. Not surprisingly, therefore, the CAPs have become a hot target in peptidomimetic research.

Many CAPs kill bacterial cells in the micromolar range by mechanisms culminating in disruption of the bacterial cell membrane [41]. The cationic peptides are first attracted electrostatically to the outer bacterial cell surface because of the presence of excess negatively charged phospholipids and glycolipids. They then invade and disrupt the membrane bilayer(s), eventually causing cell lysis. This process was captured recently in a series of time-resolved quantitative microscopy images of the human cathelicidin LL-37 attacking *Escherichia coli* cells [42]. However, CAPs can also lyse (typically at a higher concentration) mammalian cell membranes, which is a potential source of toxicity and one factor that has so far prevented their application for the treatment of systemic human bacterial infections. On the other hand, some CAPs clearly have different mechanisms of action, which do not involve membrane lysis (for recent reviews, see [36–40]).

**Figure 3:**
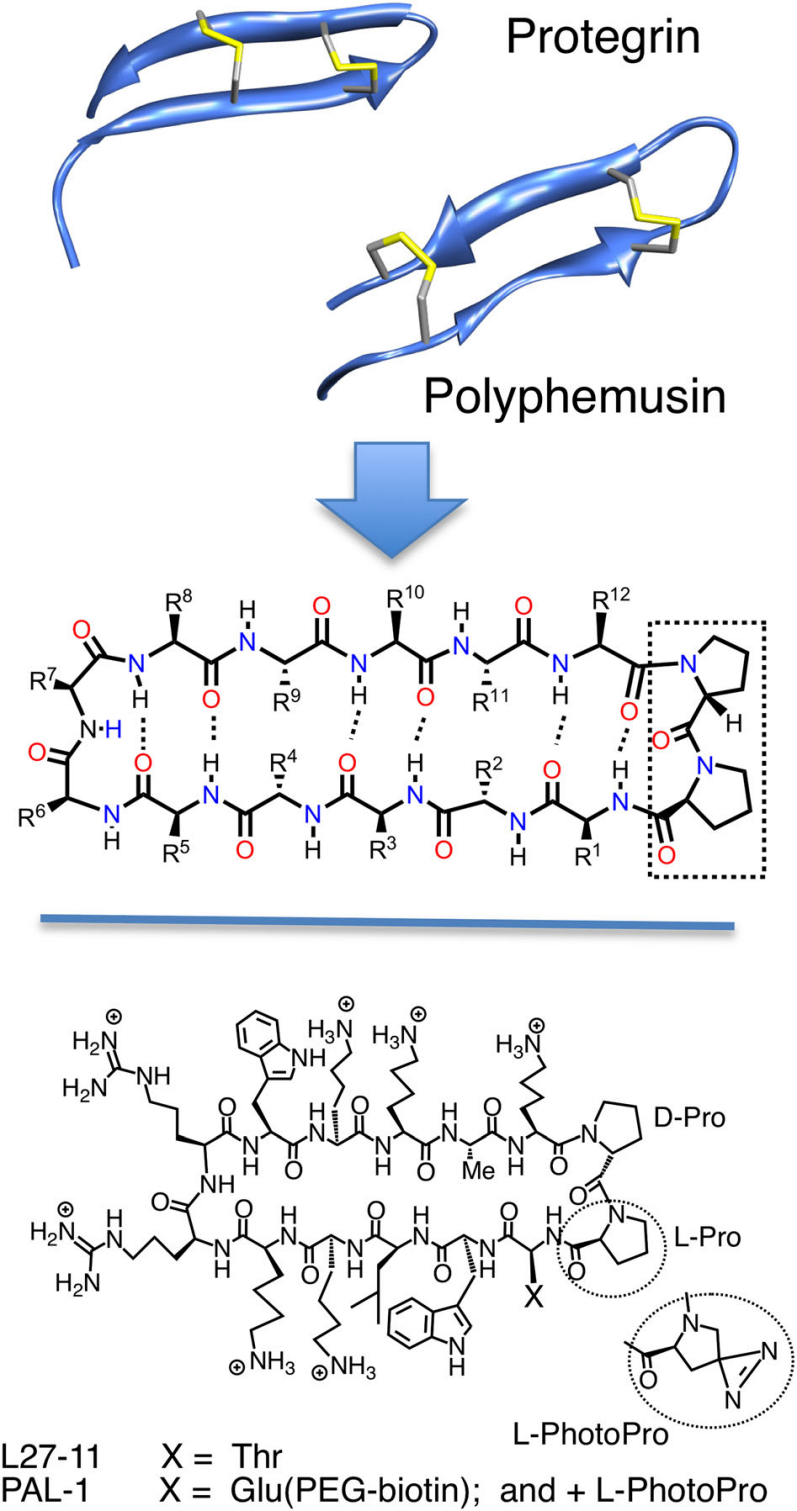
Naturally occurring *β*-hairpin-shaped CAPs provide a starting point for mimetic design. The mimetic L27-11 is a potent antibiotic acting selectively against *Pseudomonas* sp. [33]. The bacterial target of L27-11 was shown to be the OM protein LptD. The photoprobe PAL-1, which contains photoproline in place of l-proline and a biotin tag at position 1, photolabels LptD selectively.

Although a diverse array of different folded secondary structures are found among the CAPs, one group possess *β*-hairpin structures stabilized by disulfide bridges, including the protegrins, polyphemusins, tachyplesin, arenicin, and *θ*-defensin. One approach to mimic such CAPs is to exploit the properties of a hairpin-stabilizing template to generate macrocyclic PEMs with folded structures (Figure 3). The mimetics may contain sequences related to the *β*-hairpin CAPs, but without relying on the presence of constraining disulfide bridges for folding. The structural mimicry, coupled with the ease of synthesis (and therefore optimization), opened the way recently to the discovery of a new family of cyclic PEMs with a novel type of antimicrobial activity [33]. This family is represented by the cyclic peptide L27-11 (Figure 3), which shows antimicrobial activity in the *nanomolar* range specifically against Gram-negative *Pseudomonas* sp. The lead compound, L27-11, does not cause lysis of bacterial cell membranes, and only one enantiomer of the molecule has antimicrobial activity (MIC ≍ 0.01 µg/ml against *Pseudomonas aeruginosa*); the enantiomeric form is essentially inactive (MIC ≥ 32 µg/ml). The amino acid sequence of L27-11 is unrelated to that of any known naturally occurring CAP, although like the CAPs, it does contain a mix of hydrophobic (aromatic) and cationic residues. Given the potent antimicrobial activity against the important human pathogen *P. aeruginosa* and its likely novel mechanism of action, efforts have been made to develop a lead for clinical development. A related molecule, called POL7001, has a much-improved stability towards proteolysis in human plasma, due to the replacement of multiple Lys/Arg residues by diaminobutyric acid residues [33]. These substitutions do not have a large effect on antimicrobial activity but remove cleavage sites for trypsin-like proteases. Another lead called POL7080 has optimized absorption, distribution, metabolism, elimination, and toxicity properties and has recently completed successfully a human phase I clinical trial [43]. A new narrow-spectrum antibiotic targeting *P. aeruginosa* would be a welcome addition to the range of antibiotics currently available to treat serious hospital-acquired infections, as life-threatening difficult-to-treat drug-resistant strains are arising with increasing frequency both in hospitals and in the wider community [44].

The first indication of a likely mechanism of action came from photoaffinity labeling experiments with the photoprobe PAL-1 (Figure 3) and from a forward genetic screen for resistance determinants in *P. aeruginosa* [33]. Both approaches identified the same *β*-barrel OM protein LptD as a likely target. Most proteins in the OM of Gram-negative bacteria contain a *β*-barrel domain embedded in the lipid bilayer. In contrast, transmembrane proteins located in the inner membrane (IM) typically contain *α*-helical segments. Whereas transmembrane helical domains are commonly found in human membrane proteins (e.g. G protein-coupled receptors), *β*-barrel domains do not occur on the surface of human cells, although they are found in mitochondrial membranes. Many crystal structures of *β*-barrel proteins are known. For example, the OmpX protein from *E. coli* has an eight-strand *β*-barrel, whereas the *E. coli* porin OmpF has a 16-strand *β*-barrel that forms a trimer in the *E. coli* OM [45]. LptD is much larger and is predicted to contain a C-terminal *β*-barrel domain possibly containing up to 26 *β*-strands and an N-terminal domain of unknown structure that sits on the periplasmic side of the membrane. Over the past 10 years, much has been learnt about the function of LptD in *E. coli* and related Gram-negative bacteria (reviewed in [46–48]). LptD exists in a complex with the lipidated protein LptE in the OM of most Gram-negative bacteria [49], where it functions in the final step of lipopolysaccharide (LPS) translocation to the cell surface.

**Figure 4:**
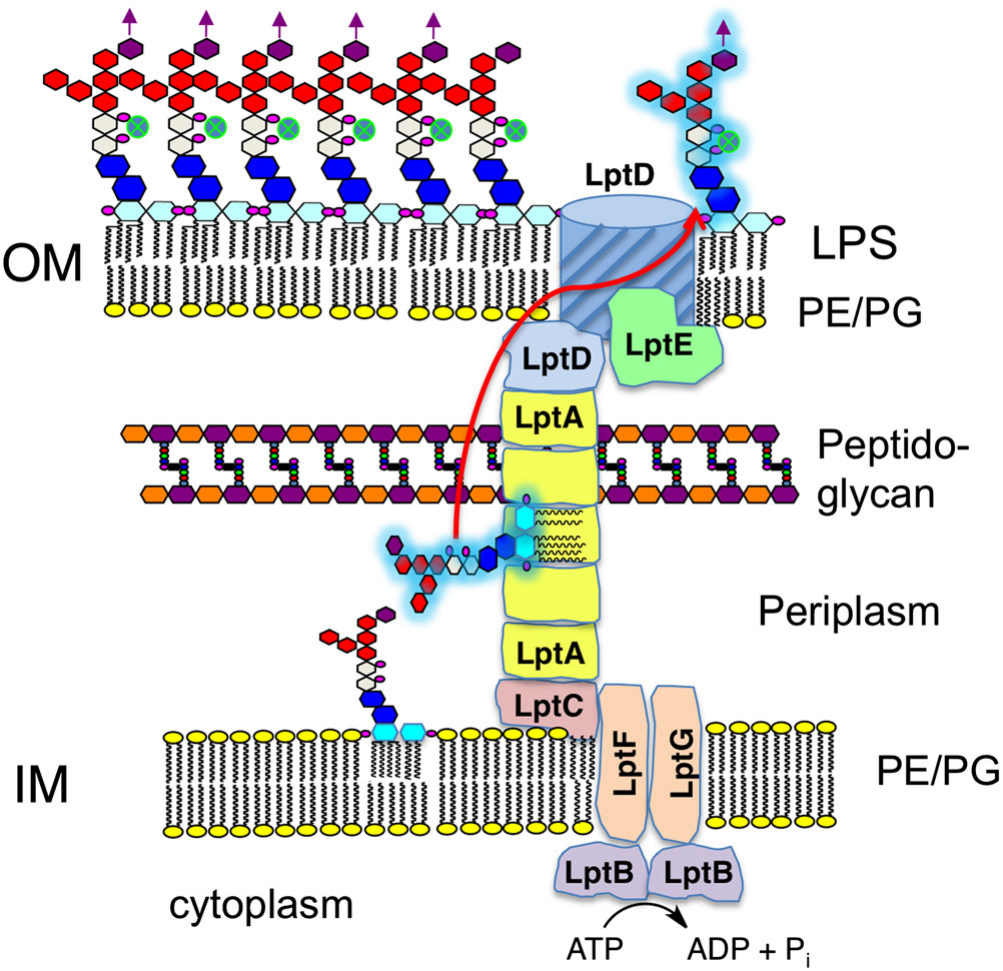
The OM protein LptD is the last component in the LPS transport pathway in Gram-negative bacteria [46–48]. LptD translocates LPS from the periplasm into the outer leaflet of the asymmetric OM.

The IM of Gram-negative bacteria is a lipid bilayer composed of phospholipids, whereas the OM is an asymmetric bilayer composed of phospholipids in the inner leaflet and LPS in the outer leaflet (Figure 4) [46]. Divalent Ca^2+^ and Mg^2+^ ions cross-link phosphate groups in LPS molecules, which strengthens considerably the OM and renders it highly impermeable to most small molecules including most antibiotics. LPS contains a hydrophobic lipid A moiety, comprising five to seven fatty acid chains connected to a disaccharide composed of *N*-acetylglucosamine. The lipid A is attached typically to an octasaccharide carbohydrate core, which in turn is often linked to a highly immunogenic O-antigen oligosaccharide [50,51]. How the LPS molecules are transported across two membranes and the intervening periplasm is only now starting to be unraveled. Seven essential LPS transport (Lpt) proteins are known to mediate this transport process [46–48]. The heteromeric ABC transporter (LptBFG) forms a complex in the IM, together with the membrane protein LptC (Figure 4). LptC then interacts with the periplasmic protein LptA, which in a head-to-tail oligomeric form likely creates a bridge across the periplasm. The LptA bridge makes contact to the N-terminal periplasmic domain of LptD in the OM [52]. This trans-envelope protein bridge provides a highway across which LPS molecules are shuttled from the IM to the OM in an ATP-dependent process [53]. The lumen of the C-terminal *β*-barrel domain of LptD appears to be at least partially occupied by LptE [49]. The LptD/E complex accepts LPS molecules from LptA in the periplasm and subsequently translocates them by an unknown mechanism into the outer leaflet. Upon exposure to L27-11, large accumulations of membrane-like material can be seen by transmission electron microscopy associated with the OM within *P. aeruginosa* cells [33]. Similar accumulations of membrane-like material are seen in *E. coli* when *lptD*, or other essential genes in the Lpt pathway, are downregulated [54,55], most likely because of the accumulation of LPS molecules in the IM.

A photolabeling experiment demonstrated that the antibiotic binds to LptD [33]. The photoprobe (PAL-1) (Figure 3) contains a photolabile amino acid l-photoproline, as well as a biotinylated Glu residue at position 1 in the hairpin. This photoprobe is still a potent antibiotic (MIC ≍ 0.05 µg/ml) against *P. aeruginosa*, despite the presence of the biotinylated side chain. After UV irradiation of cells exposed to PAL-1, a selective photolabeling of the LptD protein could be detected in membrane protein extracts. Recent studies provided further evidence that L27-11 inhibits LPS transport to the OM in *P. aeruginosa* [56]. In particular, L27-11 elicited changes to LPS structure and membrane morphology in wild-type *P. aeruginosa* cells that were identical to those seen in cells of a conditional mutant in which the *lptD* gene was downregulated. The results obtained are consistent with restricted LPS translocation to the OM, caused either by inhibition by L27-11 or by downregulation of *lptD* and its accumulation in the IM.

**Figure 5:**
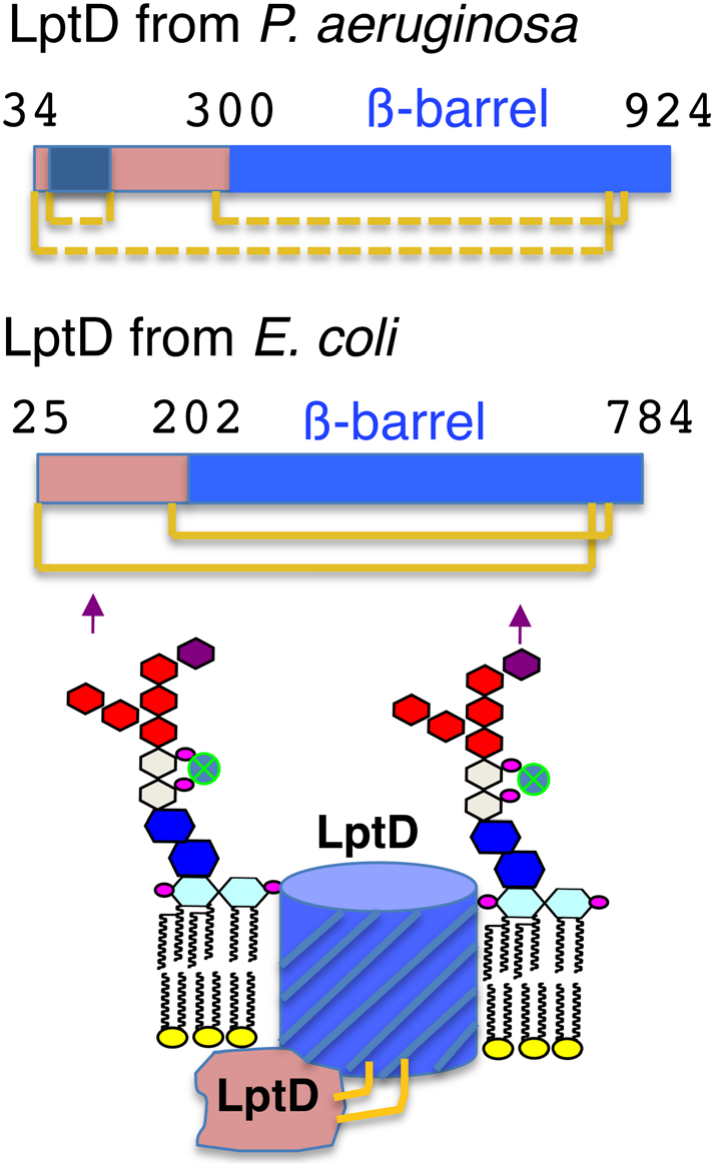
The LptD OM protein is essential in both *P. aeruginosa* (*PA*) and *E. coli* (*EC*), and the sequences share significant homology. The folded proteins lack the signal peptide (residues 1–33 in *PA* or residues 1–24 in *EC*), both contain a periplasmic domain and a C-terminal *β*-barrel domain. However, differences in sequence, length, and the number of disulfide bonds (proven in *EC*, full lines [59]; likely in *PA*, dotted lines) are seen between LptD in these organisms. These sequence differences may account for the selective action of the antibiotic L27-11 for *Pseudomonas* sp.

Recently, the folding pathway for native LptD in the *E. coli* OM has been studied in detail. LptE is required for LptD to fold correctly in *E. coli* [57]. The *β*-barrel domain is folded in the OM in a process catalyzed by the Bam machinery, a conserved complex of proteins responsible for folding and inserting *β*-barrel proteins in the OM of Gram-negative bacteria [58]. The rate-determining step in LptD/E assembly appears to be *β*-barrel folding, which is remarkably slow (20 min, corresponding to about one third of the cell cycle) [59]. After the *β*-barrel is folded, an intermediate form of LptD is a substrate for the periplasmic oxidase DsbA, which catalyzes formation of two nonconsecutive intramolecular disulfide bonds [59,60]. The *E. coli* LptD contains four Cys residues, two in the periplasmic domain (residue 25-202; Cys^31^ and Cys^173^) and two in the *β*-barrel domain (residue 203-784; Cys^724^ and Cys^725^). Interestingly, Cys residues corresponding to both Cys^173^ and Cys^725^ are conserved in >95% of over 1000 Gram-negative LptD proteins, including that from *P. aeruginosa*. The LptD from *P. aeruginosa* PAO1 strain is larger (residue 34-924) than that from *E. coli*, with much of the difference arising because of a ≍90-residue insertion within the N-terminal periplasmic domain (Figure 5). Cysteines corresponding to those found in *E. coli* LptD are present in the *P. aeruginosa* LptD (Cys^39^, Cys^270^, Cys^858^, and Cys^859^). However, two additional cysteines (Cys^49^ and Cys^134^) are present in *P. aeruginosa* LptD, flanking the ≍90-residue insert within the periplasmic domain. Although LptD is required in most Gram-negative bacteria for biogenesis of the OM, it is clear that differences in size and sequence of the protein do occur in different microorganisms, and these differences may account for the unusual selectivity of the *β*-hairpin antibiotic for *Pseudomonas* sp.

There have so far been only a few reports of CAPs that interact with OM proteins or lipoproteins in bacteria. Another example is the recently reported binding of *α*-helical CAPs to the lipoprotein OprI in *P. aeruginosa* [61]. It is a tantalizing prospect, however, that other CAPs or mimetics might be found that target, for example, LptD or other important membrane proteins in other Gram-negative human pathogens.

## Epitopes Recognized by Protective Antibodies

Vaccine development began in the 18th century with the use of whole microorganisms to generate protective immune responses in humans. However, live attenuated and inactivated whole viral and bacterial vaccines still belong to some of the most successful human vaccines in use today, e.g. oral polio, measles (standalone and in measles/mumps/rubella combination), influenza, typhoid, cholera, and Bacillus Calmette–Guérin, although there have been recurring problems associated with product contamination, genetic instability, and residual virulence. Although some early vaccines were based upon protein subunits or toxins that could be extracted from bacterial cells (e.g. diphtheria, tetanus, flu, anthrax, and rabies), it was the emergence of the tools of molecular biology that signaled a new era in vaccine development. The stimulation of protective antibody responses is still key to the success of almost all of the preventive vaccines in use today. Rather than using whole microorganisms as vaccines, identifying and producing individual molecular targets of protective antibodies, such as surface proteins or polysaccharide capsules, became an important focus of vaccine research. The challenging problem of identifying suitable surface antigens on bacterial pathogens, able to confer protection, has been approached more recently by applying large-scale genomic and proteomic technologies. Putative surface proteins on bacteria can be identified by bioinformatics, produced as recombinant proteins, and tested immunologically in a high-throughput fashion for their ability to elicit protective immune responses, an empirical approach that has been termed ‘reverse vaccinology’ [62–65]. The success of this approach in identifying protective antigens has now been demonstrated, even in some cases where conventional approaches to vaccine development had failed [66–68]. However, the use of recombinant proteins as vaccines continues to rely on the co-administration of suitable adjuvants to provide a sufficient boost to the immune system, and this remains problematic because of adjuvant toxicity [69].

Over the past few years, advances in structural biology have led to a dramatic increase in structural knowledge about how antibodies recognize vaccine antigens. The crystal structure of a pathogen-derived antigen bound to a cognate protective antibody reveals the folded antigen conformation against which a protective humoral immune response was elicited. This raises the prospect of a new era in ‘rational’ vaccine development, called structural vaccinology [2,70,71], in which this 3D structural information is used to rationally design novel and improved vaccine antigens. Several recent examples document how this approach can lead to the development of new viral and bacterial vaccine candidates [2,72,73]. The implications of this approach could be substantial, given the many pathogens for which no vaccines presently exist (e.g. malaria, HIV-1, hepatitis C, and *Staphylococcus aureus*), as well as the prospect that novel immunotherapeutic approaches might be developed to treat chronic human diseases, such as cancer.

Recently published crystal structures of protective monoclonal antibody (mAb) fragments bound to their protective epitopes are listed in Table 1. Most examples represent epitopes on viral proteins, with the largest number being from HIV-1. In many cases, new biological methods were exploited to isolate these neutralizing antibodies from human sources (e.g. by human memory B-cell immortalization [74]). Viruses typically display only a few proteins on their surface, which facilitates the identification of suitable antigens and their protective epitopes.

**Table 1:** Crystal structures of protective mAb fragments bound to their target epitopes derived from various human pathogens

Target antigen/protective monoclonal antibody	Epitope conformation	Reference	Protein Data Bank file
**HIV-1**			
Membrane proximal external region of gp41 recognized by mAb 10E8	*α*-Helical	[125]	4G6F
MPER of gp41 recognized by mAb 2F5	*α*-Helical	[126–128]	1TJG/H/I 2F5B 3D0L
MPER of gp41 recognized by mAb 4E10	*α*-Helical	[87,129,130]	1TZG 2FX7/8/9 3H3P
MPER of gp41 recognized by mAb Z13/Z13e1	*α*-Helical	[131]	3FN0
gp41 inner core HR1 trimer bound by human mAb D5	*α*-Helical	[132]	2CMR
gp41 inner core HR1 trimer bound by mAb 8066	*α*-Helical	[133]	3MA9
gp41 inner core HR1 trimer bound by human mAb HK20	*α*-Helical	[134]	2XRA
V1/V2 domain of gp120 from two HIV-1 strains recognized by mAb PG9	*β*-Hairpin glycosylated	[79]	3U4E 3U2S
V3 loop of gp120 bound to human mAb 447-52D	*β*-Hairpin	[104,105]	1Q1J 2ESX
V3 loop of gp120 bound to human mAb F425-B4e8	*β*-Hairpin	[135]	2QSC
V3 loop of gp120 bound to human mAbs 537-10D and 447-52D	*β*-Hairpin	[103]	3GHB 3GHE
V3 loop of gp120 bound to human mAbs 2557, 1006-15D, 3074, and 268-D	*β*-Hairpin	[136]	3MLR/S/T/U/V/W/X/Y/Z 3GO1
V3 loop of gp120 bound to human mAb 2219	*β*-Hairpin	[102]	2B0S/2B1H/2B1A
CD4 binding site on gp120 bound by mAb 17b	*β*-Sheet	[137]	1GC1
CD4 binding site on gp120 bound by mAb b12	Complex	[138]	2NY7
CD4 binding site on gp120 bound by mAb VRC01	Complex	[139]	3NGB
CD4 binding site on gp120 bound by mAbs VRC-PG04 and VRC03	Complex	[140]	3SE8/9
CD4 binding site on gp120 bound by mAbs b13 and F105	Complex	[141]	3IDX/Yc
**Hepatitis C virus**			
Hepatitis C virus envelope glycoprotein bound to mAb HCV1	*β*-Hairpin	[142]	4DGY 4DGV
Envelope glycoprotein and mAb AP33	*β*-Hairpin	[143,144]	4GAG/J/Y
**Influenza virus**			
H5N1 hemagglutinin stem region bound to mAb F10 (Fv)	*α*-Helical	[145]	3FKU
Hemagglutinin stem region bound to mAb CR6261	*α*-Helical	[146]	3GBN/M
Hemagglutinin head bound to mAb CH65	Complex	[147]	3SM5
Hemagglutinin stem region bound to mAb CR8020	Hairpin + loop	[148]	3SDY
Hemagglutinin H1N1 with neutralizing human mAb 2D1	Complex	[149]	3LZF
Sialic acid binding site on hemagglutinin + mAb S139/1	Complex	[150]	4GMS/T
**Respiratory syncytial virus**			
RSV F glycoprotein-derived peptide bound to motavizumab	Helix–loop–helix	[151,152]	3IXT
RSV F glycoprotein-derived peptide bound to mAb 101F	Extended	[152,153]	3O41/45
**Human metapneumovirus**			
Anti-HMPV F-neutralizing mAb DS7	Complex	[154]	4DAG
**Ebola virus**			
Ebola-neutralizing mAb KZ52	Complex	[155]	3CSY/3INU
Murine mAb 13F6-1-2 protecting against Ebola in mice	Linear	[156]	2QHR
Ebola-neutralizing mAb 14G7	Tandem *β*-hairpin	[157]	2Y6S
**Dengue virus**			
Neutralizing mouse mAb 4E11	Complex	[158]	3UZQ/V/E/3UYP
Dengue-neutralizing mAb 1a1D-2	Complex	[159]	2R29 2R69
Serotype cross-reactive and neutralizing mouse mAb 2H12	Complex/hairpin loop	[160]	4AL8/4ALA/4AM0
**Hepatitis B virus**			
Neutralizing mAb HzKR127	*β* + 3_10_ helical turns	[161]	2EH8
**Bacillus anthracis**			
Anti-anthrax protective antigen mAb M18	Complex	[162]	3ETB

How has structural knowledge of epitopes recognized by protective antibodies been used for vaccine design? One approach involves epitope grafting, using the tools of protein engineering. Here, the epitope of interest is transferred onto a new protein scaffold (perhaps more stable, or easier to produce, or more immunogenic) that can display the epitope in the correctly folded conformation. Several recent examples document how structure-based methods and modeling can allow prediction of proteins that might be useful as scaffolds for the newly grafted epitopes [72,75–83]. In some cases, however, it might still be technically difficult to produce the correctly folded protein subunit vaccine. Moreover, other nonprotective epitopes on the surface of a recombinant protein may still dominate the immune response, thereby deflecting attention away from the protective epitope and leading to a poorly effective vaccine. Also, co-administration with an adjuvant will again be required to boost immunogenicity.

An alternative approach is to exploit advances in synthetic peptide and protein engineering, which use the tools of organic and peptide chemistry for the production of folded proteins and related epitope mimetics. Conformational flexibility is one key parameter that must be addressed in the design of synthetic epitope mimetics. The use of flexible peptides as immunogens often elicits antibodies that bind weakly (≥micromolar *K*_D_) to conformational epitopes in folded proteins. However, antibodies that bind tightly (≤nanomolar *K*_D_) to an antigen are usually required to protect against infection, and their efficient production in an immune response will require the use of correctly folded epitope mimetics.

## Protein Epitope Mimetics in Vaccine Design

Many of the protective epitopes shown in Table 1 contain loop, *β*-hairpin, or *α*-helical motifs. So conformationally constrained synthetic epitope mimetics based on these structures may be useful as immunogens in vaccine design. For example, a number of different technologies for the stabilization of helical conformations in peptides have been developed (reviewed in [84]). Helical conformations can be stabilized through the insertion of amino acids with restricted conformational space, such as *α*-methylated amino acids (e.g. Aib), by side-chain cross-linking or ‘stapling’, and the use of helix caps and hydrogen-bond surrogates. Some of these approaches have been explored already in vaccine design efforts, for example, the use of Aib residues to favor helical turns [85–88], hydrazone cross-links as hydrogen bond surrogates [89–91], Freidinger-like lactams and pseudoprolines to stabilize turns [92,93], and cross-linked (or ‘stapled’) side chains to stabilize helical epitopes [87,94]. It seems likely that these technologies will continue to be refined and applied to larger synthetic protein and glycoprotein scaffolds.

**Figure 6:**
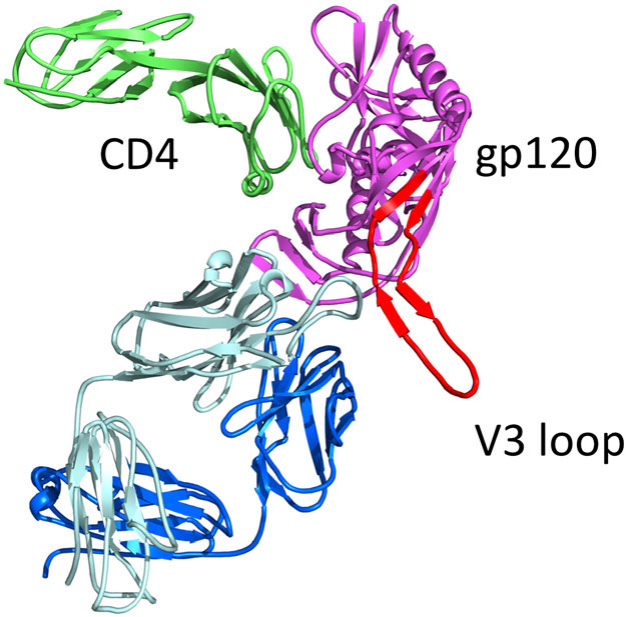
Crystal structure (Protein Data Bank 2B4C) of a complex formed by an engineered gp120 HIV-1 glycoprotein with domains from the cellular receptor CD4 and with a mAb Fab fragment [124]. The V3 loop of gp120 is in red.

*β*-Hairpin mimetics might also be very useful in synthetic vaccine design (Table 1). For example, the *β*-hairpin V3 loop is a highly immunogenic region of the HIV-1 envelope glycoprotein gp120 that becomes exposed on the viral surface once the CD4 receptor on target cells binds to the viral gp120 glycoprotein (Figure 6) [95]. The tip of the V3 loop in gp120 is then able to dock with the cellular chemokine coreceptor (CXCR4 or CCR5), which ultimately leads to virus entry into the cell. Many crystal structures are now available for neutralizing antibody fragments bound to peptides derived from the HIV-1-gp120 V3 loop (Table 1). The V3 loop has been the focus of many earlier studies in epitope mimetic design. V3-loop-derived linear peptides themselves are flexible in solution and do not adopt folded structures. The strategies used so far for loop mimetic design include macrocyclization of linear peptides [96,97], introduction of nonnatural amino acids to stabilize turn conformations [85,91–93], or introduction of one or more cross-strand disulfide bridges to stabilize loop conformations [98–100].

**Figure 7:**
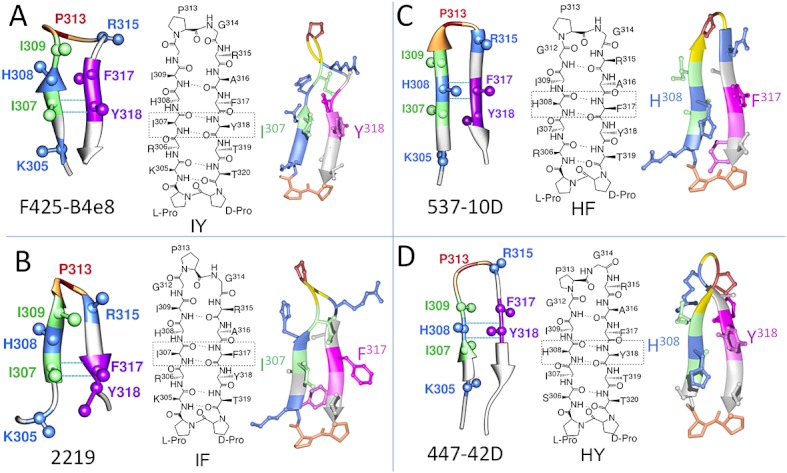
Design of four *β*-hairpin mimetics (called IY, IF, HF, and HY) based upon V3-derived peptides bound to four different neutralizing mAbs [163]. The hairpin loops have different hairpin registers in the four complexes. The hairpin registers are fixed after transfer to the d-Pro- l-Pro template. For each, the *left* side shows the bound V3 loop conformation taken from the Protein Data Bank file, and the *right* side shows one typical NMR structure of each mimetic. The template is shown in orange at the bottom of each structure.

V3 loop mimetics can also be designed by transplanting the loop sequences from gp120 onto the hairpin-stabilizing d-Pro- l-Pro template discussed earlier [22]. The d-Pro- l-Pro template can serve both to stabilize *β*-hairpin conformations and to fix the hairpin register of amino acids in HB and NHB positions. Crystal structures of V3-derived peptides bound to the mAbs F425-B4e8 [101], 2219 [102], 537-10D [103], and 447-52D [104,105] reveal the V3 loop in *β*-hairpin conformations that differ in the hairpin register. With mAb 2219, the I307 and F317 side chains point to the same side of the hairpin and occupy an HB position, whereas with mAb F425-B4e8, I307 and Y318 are the HB pair, and in the complex with 537-10D, the pair H308 and F317 are at an HB position. Four *β*-hairpin mimetics (called IY, IF, HF, and HY, Figure 7) were designed by transplanting each loop such that an HB pair is directly attached to the d-Pro- l-Pro template. ^1^H-NMR studies revealed for each mimetic an extensive network of long-range NOEs between backbone protons in cross-strand residue pairs, as well as between the side chains of residues located on the same face of the hairpin, which leaves no doubt that *β*-hairpin structures are highly populated and adopt the expected hairpin registers.

## Synthetic Virus-like Particles in Vaccine Design

The synthetic epitope mimetics described earlier are not expected to be immunogenic on their own and so require an appropriate method of delivery to the immune system. The traditional approach taken to generate immune responses against weakly immunogenic peptides and other small molecules (e.g. haptens) involves coupling to a carrier protein, such as keyhole limpet hemocyanin, and administration by subcutaneous injection together with an immunostimulatory adjuvant. Often, the effects upon peptide and protein structure of both the conjugation process and administration with either an oil-in-water-based or particulate (e.g. alum) adjuvant are unknown. These problems of antigen delivery have long been recognized and fortunately can now be addressed as a result of progress made over recent decades in understanding, at cellular and molecular levels, the mechanisms by which the immune system is activated [106].

The immune system is activated very efficiently by viruses and bacteria because they incorporate key signals that initiate an immune response. Thus, viruses and bacteria display a repetitive and closely spaced array of epitopes across their surface. Such an arrangement of epitopes is specifically recognized as foreign by B cells [107]. Multiple copies of an epitope on the pathogen may engage multiple B-cell receptors (BCRs) on individual B cells. The cross-linking of multiple BCRs at the cell surface generates a powerful signal, which initiates the process of B-cell activation and maturation [108]. In addition, the proteins in viruses and bacteria contain peptide sequences that can function as T-cell epitopes to activate the T-cell arm of the immune system, including the CD4^+^ T cells that provide crucial help to B cells and the CD8^+^ T cells required for cell-mediated immunity [109]. Furthermore, viruses are typically 20–100-nm spheres, an ideal size and shape for trafficking through the lymphatic system to the many lymph nodes (≍450) in the human body and then for presentation to B cells by follicular dendritic cells (DCs) [110]. Circulating naïve B and T lymphocytes halt in the lymph nodes, where they encounter antigens and antigen-presenting cells (APCs) draining through in interstitial fluids from peripheral tissues. Here, intact antigens are presented to B cells whereupon epitope BCR recognition can occur. APCs present T-cell epitopes as linear peptides bound to major histocompatibility complex molecules for recognition by T-cell receptors on T cells. The B and T cells become activated in a dance that finally leads to the production of antibody molecules optimized to bind the antigen [111]. Finally, viruses and bacteria contain molecules unique to themselves (called pathogen-associated molecular patterns), which the innate immune system recognizes as being foreign, using pattern recognition receptors, such as the Toll-like receptors [112,113]. The pathogens can interact directly with several types of human immunocytes containing pattern recognition receptors, including monocytes, macrophages, and myeloid and plasmacytoid DCs. These interactions induce various innate immune responses, including the production of proinflammatory cytokines, as well as lead to the maturation and migration of DCs to lymph nodes, thereby promoting adaptive immune responses.

One promising approach to vaccine delivery exploits the potent immunostimulatory properties of virus-like particles (VLPs) and related artificial nanoparticles [114–116]. VLPs may integrate the key immune stimulatory signals in one nano-sized particle, resulting in potent immunological activity [114]. VLPs are typically made of viral capsid proteins that self-assemble into particulate structures (20–100-nm diameter) closely resembling the natural viruses from which they are derived. They lack genetic material and so are noninfectious and replication incompetent. Examples of VLP-based vaccines in current use are the hepatitis B vaccine made from the surface antigen [117] and the human papillomavirus vaccine (Cervarix and Gardasil) made from the L1 surface protein [118], which both spontaneously form VLPs in solution. The hepatitis B vaccine made from the surface antigen was also the first widely used recombinant protein vaccine to be invented.

Many other VLP-based systems are now under development for use as carriers in vaccine research and development [114,115]. One idea is to engineer the capsid proteins so that protective foreign epitopes can be inserted and displayed on the surface of the particle. Of course, suitable sites in the capsid protein must be identified, into which foreign domains containing the protective epitope can be inserted and displayed on the VLP surface. The inserted domain must fold correctly, and the engineered capsid protein must still be able to self-assemble into VLPs, which is not always easy to predict. Alternatively, a chemical coupling approach can be taken to conjugate epitopes to VLPs. Apart from biotechnological VLPs, however, there is also great interest in chemical approaches to VLP-like nanoparticles for use as vaccine carriers [116].

**Figure 8:**
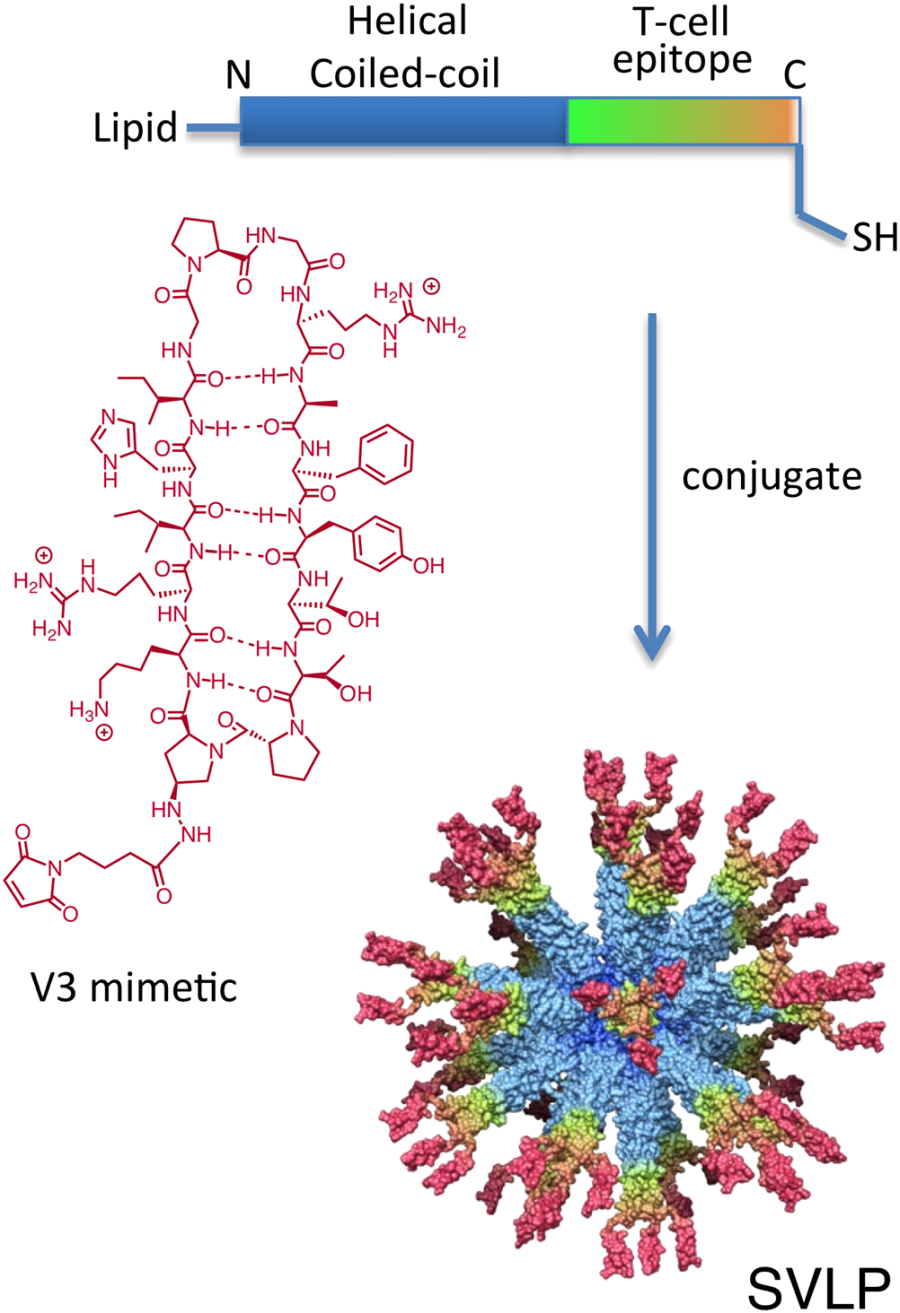
SVLPs are produced by spontaneous self-assembly from lipopeptide building blocks, in which the peptide sequence includes a coiled coil linked to a T-cell epitope. Epitope mimetics can be linked to the lipopeptide, for example, the V3 mimetic shown can be linked to a C-terminal Cys residue. The model of the resulting SVLP nanoparticle is based upon extensive biophysical characterization [22,119–121].

One chemical approach reported recently exploits the unique chemical and physical properties of designed synthetic lipopeptide building blocks, which in aqueous buffers spontaneously self-assemble into homogeneous nanoparticles in the 20–30-nm size range, called *synthetic* VLPs (SVLPs) (Figure 8) [22,119,120]. The lipopeptide building blocks include a coiled-coil sequence capable of forming a parallel trimeric helical bundle, fused to a CD4^+^ T-helper epitope. The lipid portion is typically a phospholipid (phosphatidylethanolamine) or a bacterial Toll-like receptor ligand such as Pam_2_Cys or Pam_3_Cys, which is coupled to the terminus of the peptide chain. A synthetic protein epitope mimetic can then be coupled close to the other end of the peptide chain. Self-assembly into SVLPs then occurs spontaneously in aqueous solution, driven by formation of trimeric helical bundles and then by association of multiple bundles into a micelle-like particle with the lipid chains buried in the core of the nanoparticle [120,121]. The dimensions (20–30-nm diameter) and constitution (peptide + lipid) of the nanoparticles resemble those of some natural viruses, but the SVLPs are produced by chemical synthesis. SVLPs present a dense array of about 70–80 copies of the epitope mimetic over the surface of the nanoparticle. A recent study showed that DCs bind rapidly to SVLPs, which are then internalized using multiple endocytic routes, dominated by caveolin-independent lipid raft-mediated macropinocytosis [122]. Processing then occurs more slowly by proteolytic cleavage of the lipopeptides. The processing is highly effective, however, as evidenced by the strong immune responses induced by SVLPs in animals, without need for external adjuvants [22,119,120].

Epitope mimetics can be linked to SVLPs site specifically and with high efficiency using a variety of coupling chemistries. For example, a *β*-hairpin V3 loop mimetic was coupled to SVLPs using a unique Cys residue near the C-terminus of the lipopeptide building block (Figure 8) [22]. A computer model of the resulting SVLPs shown in Figure 8 is supported by extensive biophysical data and illustrates the dense array of epitope mimetics displayed on the particle surface. The V3-SVLPs are highly immunogenic in animal models. In rabbits, high titers of V3-mimetic-specific IgG antibodies are induced by these V3-SVLPs, including antibodies that bind specifically to recombinant gp120 by ELISA [22]. The HIV-1-neutralizing activity of the affinity purified anti-V3 mimetic IgG was also tested using a whole-cell luciferase reporter-gene assay, based upon a single round infection with molecularly cloned Env-pseudotyped viruses. However, in this experiment, only infection by the laboratory MN strain was successfully inhibited by the IgG. No inhibition was seen for neutralization-insensitive tier 2 HIV-1 strains isolated from HIV-infected humans, unless the viruses were first engineered by deleting the V1V2 loop region. This result, however, is in agreement with recent studies showing that V3 loop antibodies often fail to reach their target on intact envelope trimers on the viral surface because of active shielding by the V1V2 loops, which in most cases results in only marginal or no neutralization activity [123]. New strategies that overcome this V1V2 shielding and/or that directly target protective epitopes in V1V2 are now needed [79].

Nevertheless, these studies illustrate one approach for the structure-based rational design of vaccine candidates. The approach is structure and chemistry based and should allow the design of vaccine candidates that focus the immune response on selected protective epitopes, delivered in a highly immunogenic format. The next step will be to demonstrate that a functional protective vaccine can be made using this approach. There are certainly plenty of important targets for vaccine research awaiting attention.
